# Virtual-screening workflow tutorials and prospective results from the Teach-Discover-Treat competition 2014 against malaria

**DOI:** 10.12688/f1000research.11905.2

**Published:** 2018-02-19

**Authors:** Sereina Riniker, Gregory A. Landrum, Floriane Montanari, Santiago D. Villalba, Julie Maier, Johanna M. Jansen, W. Patrick Walters, Anang A. Shelat

**Affiliations:** 1 Laboratory of Physical Chemistry, ETH Zürich, Zürich, Switzerland; 2 T5 Informatics GmbH, Basel, Switzerland; 3 Pharmacoinformatics Research Group, Department of Pharmaceutical Chemistry, University of Vienna, Vienna, Austria; 4 IMP - Research Institute of Molecular Pathology, Vienna Biocenter, Vienna, Austria; 5 Department of Chemical Biology & Therapeutics, St. Jude Children's Research Hospital, Memphis, TN, USA; 6 Department of Global Discovery Chemistry, Novartis Institutes for BioMedical Research, Emeryville, CA, USA; 7 Relay Therapeutics, Cambridge, MA, USA

**Keywords:** Malaria, neglected diseases, virtual screening, machine learning

## Abstract

The first challenge in the 2014 competition launched by the Teach-Discover-Treat (TDT) initiative asked for the development of a tutorial for ligand-based virtual screening, based on data from a primary phenotypic high-throughput screen (HTS) against malaria. The resulting Workflows were applied to select compounds from a commercial database, and a subset of those were purchased and tested experimentally for anti-malaria activity. Here, we present the two most successful Workflows, both using machine-learning approaches, and report the results for the 114 compounds tested in the follow-up screen. Excluding the two known anti-malarials quinidine and amodiaquine and 31 compounds already present in the primary HTS, a high hit rate of 57% was found.

## Introduction

Teach-Discover-Treat (TDT) is an initiative that aims to provide high-quality tutorials of important tasks in computer-aided drug discovery, in order to impact education and drug discovery for neglected diseases ^1^. The TDT steering committee consists of computational chemists from both academia and industry. To encourage the creation of high-quality tutorials by the computational chemistry community, competitions are launched with a series of different challenges, and the results/tutorials are made available through the website of the initiative ( http://www.tdtproject.org). The competitions are open to everybody. After the first successful competition in 2012 ^2^, a second competition was launched in 2014, with four challenges. In this study, we focus on Challenge 1: ligand-based virtual screening (VS) against malaria. The goal was to build a predictive model for anti-malaria activity based on a phenotypic high-throughput screen (HTS), and to use that model subsequently to select the next set of compounds for screening. In a ligand-based VS, typically no structural information of the target is available, and thus the prediction of potentially active compounds is based on the principle that similar molecules exhibit similar activity ^3^. The challenge is thereby to find an appropriate molecular description for similarity, which can depend heavily on the compound selection and/or target ^4– 7^. In recent years, machine-learning (ML) methods have emerged as an attractive tool to boost the predictive power of ligand-based VS approaches ^8– 12^.

Malaria is caused in humans by several species of the protozoan parasite *Plasmodium*. The most lethal species is *Plasmodium falciparum* (Pf), which causes organ failure and accumulates in the brain capillaries if left untreated. Malaria is still one of the most prevalent and deadly diseases in Africa, Asia and the Americas, with an estimate of 198 million cases in 2013 leading to approximately 584,000 deaths according to the 2014 world malaria report of the World Health Organization (WHO) ^13^. Recent advances in malaria research and drug discovery have been reviewed ^14– 19^. The anti-malaria drugs can be broadly classified into three groups: (i) compounds that interfere with the heme detoxification, (ii) compounds that target folate metabolism, and (iii) compounds that inhibit mitochondrial electron transport. The current standard of care for uncomplicated malaria is artemisinin-based combination therapies. Artemisinins belong to the third group of anti-malaria drugs and rapidly kill all the blood stages of the parasite, however, they are also cleared in a short time ^20^. Unfortunately, the emergence of resistant strains has become a major problem in recent years ^21, 22^, requiring the development of new and possibly orthogonal drugs. In the past, whole-cell phenotypic screening campaigns against Pf have been successful in identifying new lead compounds ^23^.

Challenge 1 of the 2014 TDT competition involved three tasks: (i) analysis of the data from a single-concentration phenotypic HTS of 305,568 compounds, including hit-list triaging and selection of compounds for a follow-up screen with EC _50_ measurement, (ii) building and validation of a predictive anti-malaria activity model, including a held-out test-set of 1056 compounds, and (iii) follow-up hit finding by applying the predictive model to rank-order a large dataset of commercially available compounds. The top 1000 molecules of this ranked list were considered further for experimental testing. For training, the challenge provided results for 305,568 compounds from the primary HTS, as well as EC _50_ data from a follow-up confirmatory screen for a subset of the compounds.

In this study, we present the results of two Workflows. Workflow 1 was the overall winner of the competition, and Workflow 2 showed the best performance on the held-out test set measured in the phenotypic Pf screen. Note that the two Workflows interpreted the challenge differently. In Workflow 1, only data from the primary HTS was used in the training of the predictive model in order to mimic the early phase of a drug discovery campaign. In Workflow 2, the EC _50_ data from the confirmatory assay was taken into account in order to improve the labelling of the training set. Each Workflow provided a ranked list of the top 1000 molecules, from which a total of 114 compounds (80 from Workflow 1 and 38 from Workflow 2, four were in common) were selected based on vendor availability for screening in a Pf phenotypic assay. Excluding the two known anti-malarials quinidine and amodiaquine and the 31 compounds already present in the primary HTS, 46 of 81 compounds were found to be active in the follow-up assay, which corresponds to a hit rate of 57%.

## Methods

The basis for the virtual screening workflows was a phenotypic high-throughput screen against Pf with 305,568 compounds, together with a confirmatory dose-response screen for 1524 compounds, which are reported in 23. The data is deposited in ChEMBL as part of the Neglected Tropical Diseases set ( ChEMBL-NTD) ^24^. The data is also available on the TDT website ( http://www.tdtproject.org/challenge-1---malaria-hts.html). In addition, an external held-out test set with 1056 molecules was provided for comparison of submissions ^25^. This dataset was generated in the laboratory of R. K. Guy in 2014, following the same procedure as described in 23, at the time of the TDT competition. Results for this held-out set are given in the Supplementary material.

## Workflow 1

**Figure 1.**
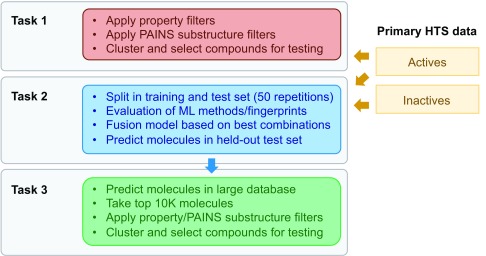
Schematic representation of Workflow 1.

The tutorial was written in the form of an IPython notebook and a series of Python scripts for the computationally demanding tasks to be executed separately. The tutorial is available on the TDT website ( http://www.tdtproject.org) and on GitHub ( https://github.com/sriniker/TDT-tutorial-2014). The tutorial makes use of a number of open-source Python libraries: the cheminformatics toolkit RDKit version 2013.09 ( http://www.rdkit.org), the machine-learning toolkit scikit-learn version 0.13 ( http://scikit-learn.org), pandas for working with data tables, and libraries for scientific computing, numpy version 1.6.2 and scipy version 0.9.0. Figures are plotted using matplotlib version 1.1.0. The components of the Workflow are shown schematically in Figure 1.

### Data preprocessing

The input for the workflow was the hit list from the phenotypic HT screen, with a classification into ‘active’, ‘inactive’, and ‘ambiguous’ compounds ^23^. From the original 305,568 compounds tested in the screen, 1528 were found to be active and 293,608 inactive. The 10,432 molecules with an ambiguous outcome were discarded.

### Task 1: Selection of 500 molecules for follow-up testing

To triage the hit list in the first task, property filters ( Table 1) based on previously described filters ^26, 27^ were applied for *in silico* post-processing of the primary HTS data, which resulted in 1512 remaining active compounds.

**Table 1.** Property filters for *in silico* post-processing of primary HTS data. These filters are used in Workflow 1.

Property	Range
Molecular weight	100–700 g/mol
Number of heavy atoms	5–50
Number of rotatable bonds	0–12
Hydrogen-bond donors	0–5
Hydrogen-bond acceptors	0–10
Hydrophobicity	-5 < logP < 7.5

Next, the active molecules were checked for potentially problematic substructures using the PAINS filters described in 28. 1225 molecules passed these filters. From these, 500 molecules had to be picked for testing in a confirmatory assay. While making this selection, a balance between the desire to have a good sampling of the chemical space covered by the primary actives and the desire to get some structure-activity relationship (SAR) information from the confirmatory assay had to be found. The compounds were therefore clustered using the Butina algorithm ^29^ based on Tanimoto similarity with a cutoff = 0.5. The Tanimoto similarity was calculated using RDKit fingerprints (a subgraph-based fingerprint similar to the Daylight fingerprint), with a maximum path length of five. 304 clusters were found, with only 40 clusters having more than five members. The cluster centers provide a set of diverse seeds. To ensure the chance to obtain information about SAR, molecules around the cluster centers were selected: Starting with the largest cluster, the five molecules most similar to the cluster center (or 50% of the cluster members if the cluster contained less than 5 molecules) were picked.

### Task 2: Prediction of anti-malarial activity for the held-out test set

Three different machine-learning (ML) models together with three different molecular fingerprints were tested for the predictive model in task 2. The ML methods were random forest (RF) ^30^, Naive Bayes (NB) and logistic regression (LR), which showed a good performance in a previous benchmarking study ^13^. The RF models were built using 100 trees, a maximum depth of 100, and minimum one sample in a leaf. As the dataset is highly unbalanced, an undersampling technique was employed for RF, i.e. for each tree a random subset of the inactives with the same size as the actives set was used. For NB and LR, the default parameters in scikit-learn were used. The fingerprints were atom pairs (AP) ^31^, RDKit fingerprint with a maximum path length of five (RDK5) and Morgan fingerprint with a radius of 2 (Morgan2) ^32^, and are described in more detail in 8. In the version of the Workflow submitted in the competition, the AP and RDK5 fingerprints were hashed to 2048 bits, and the Morgan2 fingerprints to 1024 bits. Later on we found that a fingerprint size of 4096 bits resulted in better performances due to fewer collisions. To determine which ML method/fingerprint combinations performed best and should therefore be combined using heterogeneous classifier fusion ^13^, a retrospective evaluation was performed using the primary HTS data. Classifier fusion allows the combination of the prediction of different ML models into a single prediction (see Ref. 13 for a detailed discussion). Here, all data points from the primary screen were used (i.e. none of the property-/substructure-filters discussed above were applied) as some filters may be too strict and the ML methods are rather robust to noise. The data points were randomly split 50 times into a training set (90%) and a test set (10%). A ML model was built using the training set and the molecules in the test set were ranked based on the predicted probability to be active. From the ranked list, the receiver operating characteristic (ROC) curve was calculated and subsequently the area under the ROC curve (AUC) was determined. In addition, the enrichment factor at 5% was determined. A detailed discussion of the different evaluation methods is given in 8. The results from the retrospective evaluation, averaged over the 50 repetitions, are listed in Table 2. The performance of the ML models was also compared to simple ranking by similarity, which gave a high baseline performance. Only the RF models and one LR model were able to outperform similarity ranking. Based on these results and the analysis of the diversity in the active molecules that were identified, a classifier fusion model was proposed based on RF with RDK5, RF with Morgan2 and LR with RDK5 ( Table 2). As a last step, a fusion model was trained using all data points of the primary HTS in order to obtain predictions for the held-out test set and for task 3.

**Table 2.** Evaluation results for anti-malaria activity prediction using a 90%-training and 10%-test set split for Workflow 1. The random selection was repeated 50 times and the results were averaged over the repetitions. The maximum possible EF5% value is 20.0. Fingerprints with 4096 bits were used.

Method	AUC	STD AUC	EF5%	STD EF5%
Similarity AP	0.88	0.02	13.94	0.69
Similarity RDK5	0.88	0.02	13.75	0.74
Similarity Morgan2	0.89	0.02	14.65	0.69
NB with AP	0.80	0.02	7.40	0.64
NB with RDK5	0.81	0.02	8.27	0.80
NB with Morgan2	0.85	0.02	10.42	0.98
LR with AP	0.88	0.02	12.53	0.92
LR with RDK5	0.91	0.02	14.99	0.80
LR with Morgan2	0.88	0.02	13.30	0.75
RF with AP	0.92	0.01	14.66	0.75
RF with RDK5	0.93	0.02	15.38	0.70
RF with Morgan2	0.93	0.01	15.28	0.70
Fusion model	0.93	0.01	15.75	0.73

### Task 3: Selection of 1000 new candidates from the eMolecules catalog

In task 3, the goal was to select a list of 1000 compounds from the eMolecules ( https://www.emolecules.com) catalog, with nearly 5.5 million commercially available compounds. As a first step, the molecules were filtered using the property filters described in Table 1 except logP. logP was not applied at this stage to reduce the computational cost. This resulted in approximately 4.4 million compounds. For these, molecular fingerprints (RDK5 and Morgan2) were generated with 4096 bits and the anti-malaria activity was predicted using the fusion model trained on the primary HTS in task 2. The top ranked 10,000 compounds were taken for further selection. The logP filter (see Table 1) and PAINS substructure filters were applied at this point. Filtering resulted in 7955 compounds. To select 1000 molecules from these, the following procedure was applied, which aims – as in task 1 – at striking a balance between having a good sampling of the chemical space covered by the primary actives and improving the SAR information contained in the selected molecules.

- The highest-ranked molecule is selected as first cluster center.
- Taking the next lower molecule, the similarity to the first molecule is calculated:
  ☐ If the similarity is below 0.5, the molecule is selected as a new cluster center.
  ☐ If the similarity is above 0.85 and the cluster does not contain 6 molecules yet (including the cluster center), the molecule is selected and added to the cluster.
  ☐ Else the molecule is discarded.

The procedure was continued until 1000 compounds were selected. Unfortunately, a bug in the selection step of the original tutorial resulted in the 1000 compounds being randomly selected from the top ranked 10,000 compounds (the list of 1000 compounds that would have been selected with the correct workflow are given in Supplementary Table S5). In addition, compounds already in the primary HTS used for training were not explicitly removed from the eMolecules catalog. A corrected version of the tutorial is provided on GitHub ( https://github.com/sriniker/TDT-tutorial-2014).

## Workflow 2

The tutorial is available on the TDT website ( http://www.tdtproject.org) and on GitHub ( https://github.com/sdvillal/tdt-malaria-followup). RDKit version 2013_09_2 ( http://www.rdkit.org) was used to read the SMILES strings, compute descriptors and fingerprints. Scikit-learn version 0.14 ( http://scikit-learn.org) was used to build the models.

### Data preprocessing

The input was again the original primary HTS data ^23^ with 1528 active compounds, 293,608 inactive compounds and 10,432 molecules with an ambiguous outcome. In addition, pEC _50_ data from a dose-response confirmatory screen for 1524 compounds ^23^ was taken into account. Compounds were relabeled using, when available, the confirmatory pEC _50_ data. Any compound with a pEC _50_ of at least 5 was considered positive for anti-malarial activity independent of the original classification. As a result, 296 molecules were relabeled from positive to negative; 192 molecules were relabeled from ambiguous to negative; 52 molecules relabeled from ambiguous to positive; 4 molecules were relabeled from negative to positive. The final dataset contained 1288 compounds labeled as positives, 294,092 as negatives, and 10,188 as ambiguous. Ambiguous compounds were not considered for modeling.

### Descriptors and unfolded circular fingerprints

To describe the chemical structures of the compounds, the 196 RDKit descriptors available by default were computed. This first set will be referred to as “RDKit descriptors” set. Morgan fingerprints of both extended connectivity (ECFP) and functional class (FCFP) types ^32^ were computed with a radius of up to 200 (meaning that all possible substructures are enumerated for each compound). Typically, circular fingerprints are hashed and folded to a fixed size, but this may lead to collisions, i.e. two different substructures are hashed to the same bit in the folded fingerprint. To avoid this problem, folding was not used in Workflow 2. All the existing substructures were saved as SMARTS strings and uniquely encoded by a large bitset containing all substructures occurring in the training set. The unfolded ECFP and FCFP fingerprints were appended together in one vector. By construction of the fingerprints, there is a large amount of redundancy in the data. Duplicated features (i.e. having the same presence and absence pattern for all compounds of the data) were removed, keeping only one example. This procedure removes some of the redundancy that can negatively affect the interpretability and stability of linear models. However, it was found later that it is not relevant for performance. There were a total of 2,351,460 different SMARTS substructures in the training set, which was reduced to 1,265,410 substructures after removal of duplicates. Note that there are a total of 41,571,668 substructures present in all molecules used in the competition (including the eMolecules collection). This means that approximately 39 million substructures were not seen by the models during training. Thus, folding of fingerprints can be problematic due to collisions between “unseen” features with “seen" features. It is therefore advisable to keep track, whenever possible, of the set of features seen in the training set before folding, and to remove unknown features in test instances before folding. Supplementary Figure S5 shows the extent of the collisions problem at different maximum radii used in the circular fingerprints.

### Model building, validation and selection

Random forests ^29^ and extremely randomized trees ^33^ of 10, 20, 50, 100, 500, 1000, 2000, 4000 and 6000 trees were computed on the 196 RDKit descriptors set, using multiple random seeds. Both methods use bagging to select instances for building each tree. As a result, for each individual tree, some instances were not used for training and are referred to as “out-of-bag”. These instances can be used for an unbiased estimate of the prediction error, instead of performing a computationally expensive cross-validation. Therefore, the out-of-bag scores were used as a measure of the quality of the models, and AUC, accuracy and enrichment at 5% were computed from these scores. The ensemble of trees with 6000 trees gave the best results and was therefore selected for deployment (i.e. used for the computation of the final scores for the unlabeled datasets).

After a first exploration of multiple parameters for logistic regression on the fingerprint set by cross-validation ( Supplementary Figure S3 and Supplementary Figure S4), the following parameters for building the models were chosen: a penalty of l1 or l2, a regularization parameter C of 1 or 5, a default tolerance of 0.0001, and the fingerprints were kept unfolded. Note that despite the weak regularization and large number of features, the logistic regression models were robust against overfitting and performed well. Cross-validation was computed for 3, 5, 7 or 10 folds with five different seeds each. For each fold, the AUC and enrichment at 5% were computed. When a fold reached an AUC below 0.88, then the rest of the cross-validation was skipped and the next model was built.

The best models among the many logistic regressions models for which all folds could be completed were the ones with a penalty of l1 and C of 1 and an average AUC over all folds over 0.92; as well as those with a penalty of l2 and C of 5 and an average AUC over all folds over 0.93. These particular models were selected for deployment (i.e. used for the computation of the final scores for the three tasks).

### Task 1: Selection of 500 molecules for follow-up testing

The first task involved the selection of 500 molecules from the primary HTS set with promising activity for follow-up confirmatory measurements. For this, the predictions of the deployment models were combined by plain averaging of the model scores. Note that this corresponds to model fitting scores, since the screening set is the training set used for building the deployment models. The 500 molecules with the highest average scores were selected for the follow-up testing.

### Task 2: Prediction of anti-malarial activity for the held-out test set

In 1992, Wolpert introduced the concept of stacked generalization ^34^ to combine different models and boost the predictive power of the resulting ensemble. Here, feature-weighted linear stacking was used to combine our deployment models ^35^. For this, a linear regression was trained using the average out-of-bag scores (for the ensemble of trees models) and the average cross-validation scores (for the logistic regression models) as independent variables, and antimalarial activity as dependent variable. The resulting linear combination of models was applied to obtain the final score for the 1056 compounds of the held-out test set. Note that the linear combination placed substantially more weight on the tree models (coefficient 1.07) than on the logistic regression models (coefficient 0.07), which led to a lower performance on the held-out test set compared to that of the logistic regression models alone ( Supplementary Table S2). This could have been avoided by using part of the training set as external test set.

### Task 3: Selection of 1000 new candidates from the eMolecules catalog

For the selection of new candidates, the same feature-weighted linear stacking as described for Task 2 was used. The resulting linear combination of individual model scores was applied to obtain the final score for the compounds of the eMolecules catalog ( https://www.emolecules.com). The 1000 top-scoring compounds were selected as new candidates for further anti-malaria screening. Compounds already present in the primary HTS and the confirmatory screen used for training were not explicitly removed from the eMolecules catalog.

## Final selection process

From the two lists of 1000 new candidates, 114 molecules were selected for testing in a follow-up assay based on availability at vendors who agreed to be TDT sponsors. The set included two known anti-malarials quinidine (proposed by Workflow 1) and amodiaquine (proposed by Workflow 2). Compounds that were already in the primary HTS and the confirmatory screen provided by the TDT challenge were not removed.

## Experimental procedures

The potency of new candidates was determined as reported earlier ^23^. *Plasmodium falciparum* strain 3D7 was acquired from the Malaria Research and Reference Reagent Resource Center (MR4, catalog #MRA-102). Briefly, asynchronous parasites were maintained in culture based on the method of Trager ^36^. Parasites were grown in presence of fresh group O-positive erythrocytes (Key Biologics, LLC, Memphis, TN) in Petri dishes at a hematocrite of 4–6% in RPMI based media (RPMI 1640 supplemented with 0.5% AlbuMAX II, 25 mM HEPES, 25 mM NaHCO _3_ (pH 7.3), 100 µg/mL hypoxanthine, and 5 µg/mL gentamycin). Cultures were incubated at 37°C in a gas mixture of 90% N _2_, 5% O _2_, 5% CO _2_. For IC _50_ determinations, 20 µl of RPMI 1640 with 5 µg/ml gentamycin were dispensed per well in an assay plate (Corning 384-well microtiter plate, clear bottom, tissue culture treated, catalog no. 8807BC). An amount of 60 nl of compound, previously serial diluted in a separate 384-well white polypropylene plate (Corning, catalog no. 8748BC), was dispensed to the assay plate by hydrodynamic pin transfer (FP1S50H, V&P Scientific Pin Head) and then an amount of 20 µl of a synchronized culture suspension (1% rings, 4% hematocrite) was added per well, thus making a final hematocrite and parasitemia of 2% and 1%, respectively. Assay plates were incubated for 72 h, and the parasitemia was determined by a method previously described ^37^. An amount of 10 µl of the following solution in PBS (10X Sybr Green I, 0.5% v/v triton, 0.5 mg/ml saponin) was added per well. Assay plates were shaken for 1 min, incubated in the dark for 90 min, then read with the Envision spectrophotomer at Ex/Em of 485 nm/535 nm.

EC _50_ values were calculated using a four-parameter logistic equation as described previously ^23^. Compounds were arrayed in ten concentrations, varying from approximately 10 µM to 5 nM, and the R drc package was used to fit the observed response to the four-parameter Hill equation ^38^. The purity of all compounds was determined by UPLC (UV and ELSD purity average) and results from any compound with a purity below 95% were not reported.

### Analysis

Morgan2 fingerprints ^32^ and Tanimoto similarities were calculated using the RDKit. The scaffolds in the set of newly tested compounds were determined using the Bemis-Murcko algorithm ^39^.

## Results

### Held-out test set

The external held-out test set of the TDT challenge consisted of 101 actives and 955 inactives. The performances of the ML models of Workflow 1 and Workflow 2 on the held-out test set (1056 molecules) are given in Table 3. For Workflow 1, the results using fingerprints with 1024/2048 bits or with 4096 bits are reported. Note that the maximum possible EF5% for the held-out test set is 10.5 (as the fraction *χ* = 0.05 is smaller than the ratio of actives to inactives ^8^), whereas it is 20.0 for the primary HTS dataset. Workflow 2 gave the best performance for the held-out test set from all five submissions to this TDT challenge. For Workflow 1, the version using 1024/2048 bits was the one submitted to the TDT challenge. Later, it was found that a substantial amount of collisions due to hashing occurred in the short fingerprints, which affected the performance. Using longer fingerprints (i.e. 4096 bits), the performance could be improved and was found to be similar to that of Workflow 2. This highlights the resistance to noise of the ML methods used, since in Workflow 1 the false positives in the primary data were included. In Workflow 2, these false positives were corrected using the information from the confirmatory screen.

**Table 3.** Evaluation results for anti-malaria activity on the held-out test set (1056 molecules). Predictions were obtained using the fusion models of Workflow 1 and the linear combination of model scores of Workflow 2. The maximum possible EF5% value is 10.5.

Method	AUC	EF5%
Workflow 1 - Fusion model (1024/2048 bits)	0.74	2.76
Workflow 1 - Fusion model (4096 bits)	0.75	4.75
Workflow 2	0.79	4.34

**Figure 2.**
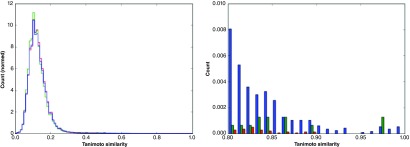
Similarity distributions for the molecules in the primary HTS and the held-out test set. Normalized Tanimoto similarity distribution using a Morgan2 fingerprint ^32^ between all possible pairs of molecules within the set of actives of the primary HTS (blue), and between this set and the set of actives (green) and of inactives (red) of the held-out test set. The full distributions (left) and the slice between 0.8 and 1.0 similarity (right) are shown. For the primary screen, the original classification into actives and inactives was used ^23^. For the held-out test set, a cutoff of 10 μM was employed.

For both Workflows, the AUC and the EF5% values were found to be substantially lower for the held-out test set compared to the values for the 10%-test split in Table 2. Although the size distribution and flexibility of the compounds in the different sets were similar ( Table 4) and the similarities within and across the datasets were generally low (left panel in Figure 2), there are slightly more highly similar compounds among the actives of the primary HTS (as in the original classification ^23^) than between those and the actives in the held-out test set (right panel in Figure 2). In addition, there were some highly similar compounds between the actives in the primary HTS and the inactives in the held-out test set.

**Table 4.** Properties of the molecules in the primary HTS and in the held-out test set. The compounds in the primary HTS were split into 1528 actives and 293,606 inactives. The compounds in the held-out test set were split into 101 actives and 955 inactives. For the primary screen, the original classification into actives and inactives was used ^23^. For the held-out test set, a cutoff of 10 μM was employed.

Dataset	Median molecular weight [g/mol]	Standard deviation of the molecular weight [g/mol]	Median number of rotatable bonds	Standard deviation of the number of rotatable bonds
Actives in primary HTS	394.0	69.4	5.0	2.2
Inactives in primary HTS	373.1	67.8	5.0	2.0
Actives in held-out test set	387.2	76.2	5.0	1.9
Inactives in held-out test set	374.1	80.6	5.0	2.0

### Prospective phenotypic screen

**Figure 3.**
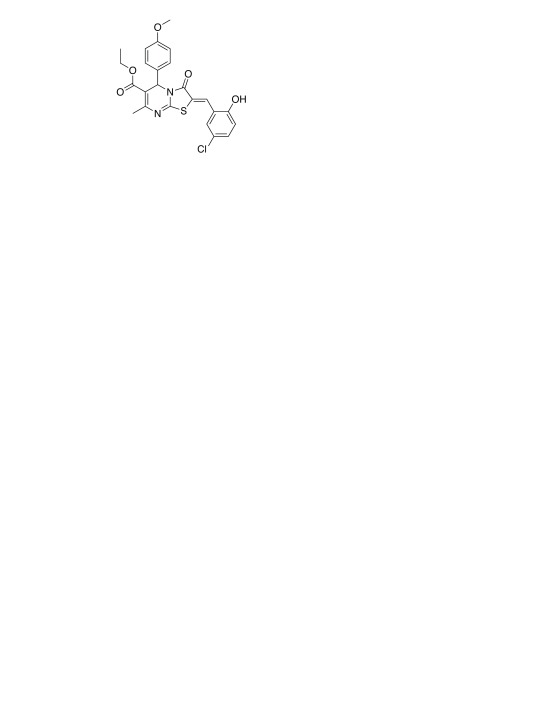
Compound SJ000154494 (EC _50_ = 0.44 µM as measured in this study).

From the combined set of 2000 candidates predicted by Workflow 1 and Workflow 2, 114 were tested in a follow-up assay (80 from Workflow 1 and 38 from Workflow 2, four compounds were predicted by both Workflows). The identifiers, SMILES, EC _50_ values and raw data for all 114 compounds are given in the Supplementary material. Of these, two were known anti-malarials (quinidine and amodiaquine) selected as positive control. In addition, 31 compounds (six from Workflow 1 and 28 from Workflow 2, three were in common) were already present in the primary HTS and confirmatory screen provided by the TDT challenge, as such molecules were not explicitly removed from the eMolecules catalog before the virtual screen ( Supplementary Table S1). One of these compounds, SJ000154494 ( Figure 3, EC _50_ = 0.44 µM as measured in this study) was found inactive in the previous primary screen and confirmatory screen ^23^, which was likely a false negative in the latter screen because dose-response testing immediately following the primary screen was done using compounds from stock solutions ranging in age, whereas the current experiment was performed on fresh powder.

The results for the remaining 81 new compounds and the two known anti-malarials are listed in Table 5. A list of all 114 compounds, including SMILES is provided as a separate file in the Supplementary material. Partially active or single-point active molecules were counted as inactives. As the list of 1000 compounds in Workflow 1 was randomly selected from the top 10,000 ranked compounds in the eMolecules database, the ranks in the latter list are also reported in Table 5. From the nine new molecules proposed by Workflow 2, only two were not in the top 10,000 list from Workflow 1, indicating that the two approaches pick generally similar features but do not score them in the same manner. To quantify the amount of similarity, the Tanimoto similarity between the (true) top 1000 compounds of Workflow 1 and the most similar molecule in the top 1000 set of Workflow 2 was calculated ( Supplementary Figure S1). 38% of the molecule pairs have a similarity of 1.0. Of the 81 new compounds, 46 were found to be active, resulting in an overall hit rate of 57%. In more detail, Workflow 1 gave a hit rate of 52% and Workflow 2 a hit rate of 100%. Due to the small number of compounds tested, we cannot judge if this difference in hit rate is significant. As the TDT initiative relies on contributions of compounds, a more systematic assessment is outside the scope of this effort. Interestingly, the most active compounds were ranked rather low in the top-10,000 list of Workflow 2 and the top-10,000 list of Workflow 1 compared to the other molecules tested, which emphasizes again that it is important in ligand-based VS to pick the compounds for follow-up testing relatively broadly from the top fraction.

**Table 5.** Results from the follow-up assay for 83 compounds. The columns are as follows: EC _50_ values, the final scores (active or inactive), and the ranks in the Workflows 1 and 2. Partially active or single-point active compounds were considered inactives (marked by italic font). ChEMBL-NTD datasets: Novartis-GNF Malaria Box (N) ^40^, St. Jude Children's Research Hospital Dataset (J) ^24^, GSK TCAMS (G) ^41^, DNDi HAT set (D). Compounds marked with (P) were tested in PubChem assays.

Identifier	EC _50_ [μM]	Score	Proposed by Workflow	Rank (top 10'000) Workflow 1	Rank (top 10'000) Workflow 2	Known Datasets
SJ000110703	0.025	Active	2	3907	853	Amodiaquine
SJ000285572	0.060	Active	1	6589	-	Quinidine
SJ000866784	0.099	Active	2	4544	931	
SJ000866752	0.14	Active	2	3108	826	
SJ000866753	0.18	Active	1	5240	4647	
SJ000866807	0.20	Active	1	4337	-	
SJ000361770	0.28	Active	2	3394	952	
SJ000866781	0.29	Active	1	3299	5804	
SJ000866764	0.39	Active	2	2174	720	N, P (active)
SJ000866760	0.72	Active	1	1739	2779	
SJ000866797	0.76	Active	2	-	868	P (anti-malaria: AID504832, AID504834) (active)
SJ000866778	0.77	Active	2	-	984	
SJ000866810	0.84	Active	2	974	100	N, J, G (active)
SJ000866811	0.92	Active	1	6197	-	P (not anti-malaria)
SJ000866815	0.98	Active	2	9752	569	P (anti-malaria: AID504382) (active)
SJ000866767	1.1	Active	1	4262	-	
SJ000866780	1.2	Active	1	688	-	
SJ000866773	1.3	Active	1	5138	-	
SJ000866792	1.5	Active	1	7129	-	
SJ000866800	1.9	Active	1	5205	3857	N (active)
SJ000377329	2.0	Active	1	6068	6930	
SJ000866786	2.3	Active	1	5832	-	
SJ000364456	2.4	Active	1	6073	-	
SJ000866779	3.2	Active	1	3069	-	D (inactive)
SJ000866794	3.2	Active	1	3935	1998	
SJ000866757	3.3	Active	1	6813	-	
SJ000866813	4.1	Active	1	3613	4593	
SJ000866809	4.3	Active	1	2603	-	
SJ000377299	4.4	Active	1	6318	-	
SJ000866777	4.6	Active	1	4159	-	
SJ000866750	5.4	Active	1	6016	-	
SJ000866789	7.8	Active	1	6198	-	
SJ000866790	8.2	Active	1	2624	-	
SJ000866755	8.4	Active	1	5923	-	
SJ000399327	9.2	Active	1	4383	8388	P (anti-malaria: AID504832, AID504834) (active)
SJ000866806	9.2	Active	1	5850	-	
SJ000866759	9.3	Active	1	7004	-	
SJ000866747	9.5	Active	1	4303	-	
SJ000866799	9.5	Active	1,2	3852	989	D (active)
SJ000866766	9.7	Active	1	3614	-	
SJ000866749	10.0	Active	1	3942	-	
SJ000866793	10.0	Active	1	4191	-	
SJ000866768	12.0	Active	1	2939	5006	
SJ000866762	12.0	Active	1	3282	-	
SJ000866788	14.0	Active	1	1166	5169	
SJ000866798	14.0	Active	1	6416	-	
SJ000420481	17.0	Active	1	3378	-	
SJ000866776	18.0	Active	1	61	-	
SJ000866769	3.7	*Inactive*	1	5649	-	
SJ000866796	4.6	*Inactive*	1	3684	-	
SJ000866804	6.1	*Inactive*	1	1366	-	
SJ000866765		*Inactive*	1	31	-	
SJ000866771		*Inactive*	1	4272	-	
SJ000866783	7.2	*Inactive*	1	5414	-	P (anti-malaria: AID504832, AID504834) (inactive)
SJ000866802	7.9	*Inactive*	1	2598	-	
SJ000866808	11.0	*Inactive*	1	4051	-	
SJ000866748	11.0	*Inactive*	1	6407	-	
SJ000866785	19.0	*Inactive*	1	5202	-	
SJ000866751	6.0	*Inactive*	1	880	-	
SJ000389261	6.0	*Inactive*	1	7634	-	P (anti-malaria: AID504832, AID504834) (inactive)
SJ000866758	8.8	*Inactive*	1	6525	-	
SJ000866746	15.0	*Inactive*	1	6110	-	
SJ000866782		Inactive	1	23	-	
SJ000866803		Inactive	1	2269	-	
SJ000866805		Inactive	1	2468	-	
SJ000388303		Inactive	1	2630	-	
SJ000866770		Inactive	1	2879	-	
SJ000866801		Inactive	1	3588	-	
SJ000866772		Inactive	1	3600	-	
SJ000866775		Inactive	1	3948	-	
SJ000866763		Inactive	1	4385	-	
SJ000866761		Inactive	1	4405	-	
SJ000866745		Inactive	1	5167	-	
SJ000866791		Inactive	1	5376	-	
SJ000866812		Inactive	1	5792	-	
SJ000866795		Inactive	1	7149	-	
SJ000866756		*Inactive*	1	852	-	
SJ000866754		*Inactive*	1	1257	-	
SJ000866814		*Inactive*	1	2606	-	
SJ000394036		*Inactive*	1	3073	-	
SJ000866774		*Inactive*	1	3858	-	
SJ000866787		*Inactive*	1	5124	5826	
SJ000391199		*Inactive*	1	6194	-	

**Figure 4.**
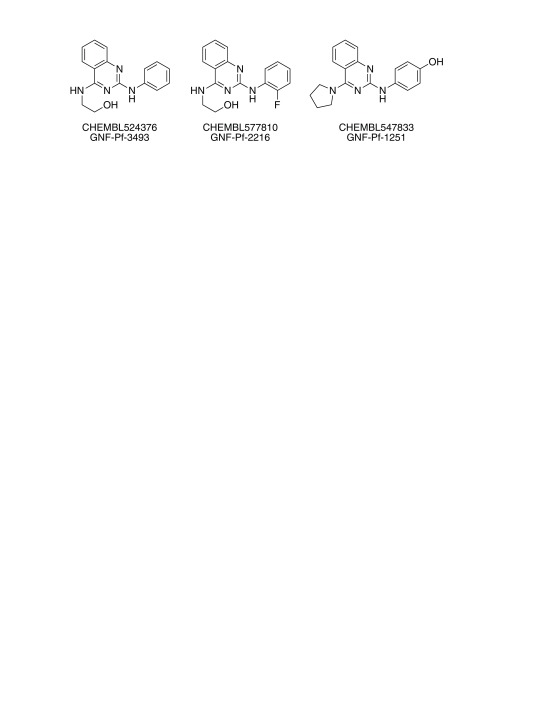
Compounds from the Novartis-GNF Malaria Box ^40^, with an aminopyrimidine-type scaffold. These compounds are similar to the group of compounds predicted by Workflow 1 with the same scaffold ( Table 6).

For Workflow 1, six of the 73 new compounds were tested previously in anti-malaria activity assays found in ChEMBL-NTD ( https://www.ebi.ac.uk/chemblntd/) and PubChem ( https://pubchem.ncbi.nlm.nih.gov) and three of them were found to be active. Three main scaffolds covered 25 of the 73 compounds: thiazolidin-4-one-type, 8-hydroxyquinoline-type, and aminopyrimidine-type scaffolds ( Table 6). The compounds with the thiazolidin-4-one-type scaffold were the largest group. The scaffold can be seen as a variation of compound SJ000154494 ( Figure 3), but the compounds in this group were mostly inactive. In addition, the scaffold may be a potential PAINS substructure due to its similarity with rhodanine, although it is currently not part of the filters ^28^. The 8-hydroxyquinoline scaffold is a phenolic Mannich base, which is a PAINS substructure. The most interesting scaffold is the aminopyrimidine-type with a second *N*-alkyl substituent instead of a known *N*-aryl substituent. The most active compound of this series, SJ000866807, exhibits a good ligand efficiency with an EC _50_ of 0.2 μM and a molecular weight of only 266 g/mol. From this series of compounds only one (SJ000866811) was listed in PubChem, but this was in an assay for anti-cancer activity (AID 743276). However, similar compounds were previously reported in the Novartis-GNF Malaria Box ^40^ ( Figure 4).

**Table 6.** The three main scaffolds present in the 73 compounds predicted by Workflow 1. ChEMBL-NTD datasets: Novartis-GNF Malaria Box (N) ^40^, St. Jude Children’s Research Hospital Dataset (J) ^24^, GSK TCAMS (G) ^41^, DNDi HAT set (D). Compounds marked with (P) were tested in PubChem assays.

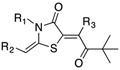					
**Identifier**	**R1**	**R2**	**R3**	**EC _50_ [μM]**	**Known Datasets**
SJ000388303	H	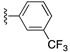	H	-	
SJ000391199	H	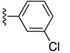	H	-	
SJ000389261	H	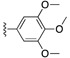	H	6.0	P (anti-malaria: AID504832, AID504834)
SJ000394036	H	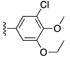	H	-	
SJ000866774	H	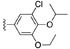	H	-	
SJ000866791	H	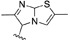	H	-	
SJ000866759	H	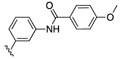	H	9.3	
SJ000866776	H	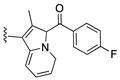	H	18.0	
SJ000866756	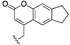	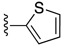	H	-	
SJ000866814	Phenyl-	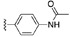	Cyano-	-	
SJ000866809	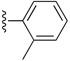	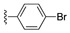	Cyano-	4.3	
SJ000866805	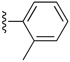	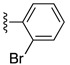	Cyano-	-	
SJ000866804	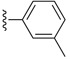	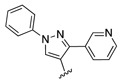	Cyano-	6.1	
SJ000866802	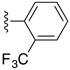	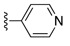	Cyano-	7.9	
SJ000866801	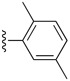	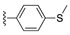	Cyano-	-	
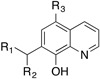					
**Identifier**	**R1**	**R2**	**R3**	**EC50 [μM]**	**Known Datasets**
SJ000866799	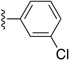	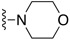	H	9.5	D
SJ000866771	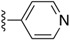	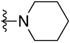	H	-	
SJ000866779	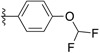	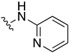	H	3.2	D
SJ000866777	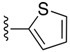	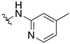	H	4.6	
SJ000866800	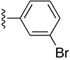	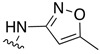	H	1.9	N
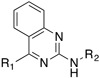					
**Identifier**	**R1**	**R2**		**EC50 [μM]**	**Known Datasets**
SJ000866807	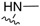	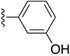		0.20	
SJ000866760	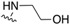	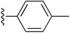		0.72	
SJ000866811	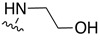	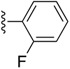		0.92	P (not anti- malaria)
SJ000377329	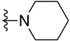	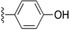		2.0	
SJ000377299	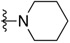	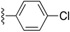		4.4	

**Figure 5.**
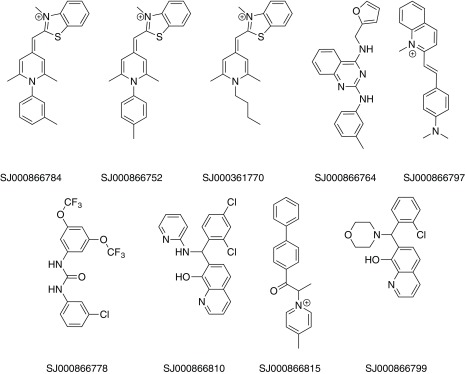
Nine compounds proposed by Workflow 2. The molecules are ordered by decreasing activity.

**Figure 6.**
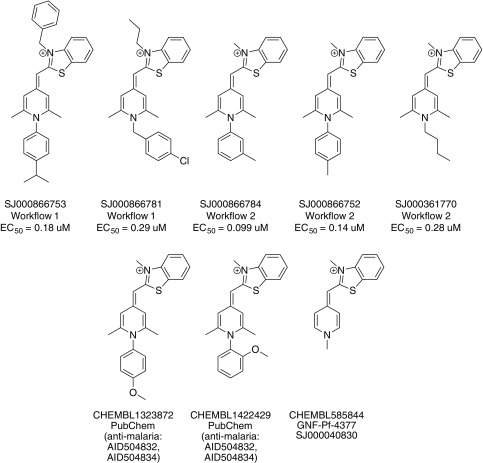
Compounds with a benzothiazole scaffold. (Top): Compounds predicted by Workflow 1 and Workflow 2. (Bottom): Compounds that are actives from PubChem, Novartis-GNF Malaria Box ^40^ and St. Jude Children’s Research Hospital ^24^.

The nine new compounds proposed by Workflow 2 are shown in Figure 5. Five of them had been tested active previously in one of the ChEMBL-NTD assays or in PubChem assays for anti-malaria activity. Two compounds (SJ000866810 and SJ000866799) have the same 8-hydroxyquinoline-type scaffold as in Workflow 1, and one compound (SJ000866764) has a similar aminopyrimidine-type scaffold. Among the most active compounds predicted by both Workflows was a series of molecules with a benzothiazole scaffold ( Figure 6). Compounds with a similar scaffold were tested previously in PubChem assays for anti-malaria activity or are part of the ChEMBL-NTD datasets. Compound SJ000040830 showed also high anti-leishmanial activity ^23^. There may be, however, potential PAINS issues with this scaffold, although not covered by the current PAINS filters, as the extended π-system may act as Michael-like acceptor.

## Conclusions

The use of ligand-based VS based on results from a primary HTS to select new, potentially active compounds for testing is a common task in drug discovery. Here, we presented two detailed Workflows using open-source tools for educational purposes, and report the application of these Workflows for the identification of anti-malarial compounds as part of the 2014 TDT challenge. Information from a previous primary HTS performed at the St. Jude Children's Research Hospital (and a confirmatory screen in case of Workflow 2) was used for training. Of the 2000 compounds proposed by the Workflows, 114 were selected for follow-up testing based on availability. Excluding the two known anti-malarials quinidine and amodiaquine and the 31 compounds already present in the primary screen, 46 out of 81 new compounds were found to be active, which corresponds to a high hit rate of 57% and shows that the machine-learning methods in the presented Workflows both successfully identified scaffolds with anti-malaria activity. There was a good agreement between the two Workflows in the general scaffolds that were identified, even though the exact compounds and rankings were not the same. The most interesting group of compounds in the tested set contains an aminopyrimidine-type scaffold with a second *N*-alkyl substituent instead of a known *N*-aryl substituent. In particular, the most active compound SJ000866807 of this series shows good ligand efficiency.

## Data and software availability

The tutorials are available on the TDT website ( http://www.tdtproject.org/2014-tutorials.html) and on GitHub ( https://github.com/sriniker/TDT-tutorial-2014 and https://github.com/sdvillal/tdt-malaria-followup). Both tutorials use only freely available software as specified above. The data from the primary HTS and confirmatory dose-response assay used in the TDT competition are available on the TDT website ( http://www.tdtproject.org/challenge-1---malaria-hts.html) and are also deposited in ChEMBL, as part of the Neglected Tropical Diseases set ( ChEMBL-NTD). The identifiers, SMILES, EC _50_ values and raw data for the held-out test set ^25^, as well as for the 114 compounds tested in this study, are given in the Supplementary material.

## Supplementary material

Supplementary Table S1: Identifiers, EC _50_ values, final scores and ranks in the Workflows 1 and 2 for the 31 tested compounds that were part of the primary HTS screen.

Supplementary Table S2: Evaluation results for anti-malaria activity on the held-out test set (1056 molecules) for different models of Workflow 2.

Supplementary Table S3: Identifiers, SMILES, EC _50_ values and raw data for the 1056 molecules in the external held-out test set

Supplementary Table S4: Identifiers, SMILES, EC _50_ values and raw data for the 114 molecules tested in this study

Supplementary Table S5: Identifiers, SMILES, Best Rank and Probability for the 1000 molecules that would have been selected by the corrected Workflow 1

Supplementary Figure S1: Distribution between the (true) top 1000 compounds of Workflow 1 and the most similar molecule in the top 1000 list of Workflow 2.

Supplementary Figure S2: Influence of the number of trees on the tree models performance (AUC and enrichment at 5%).

Supplementary Figures S3: Parameter exploration for the logistic regression models of Workflow 2 using the area under the ROC curve (AUC).

Supplementary Figures S4: Parameter exploration for the logistic regression models of Workflow 2 using the enrichment at 5%.

Supplementary Figure S5: Number of molecules in the eMolecular catalogue as a function of the number of features in the fingerprint not present in the training set.
