# Supplementary material for: Virtual-screening workflow tutorials and prospective results from the Teach-Discover-Treat competition 2014 against malaria
Source: F1000Res. 2018 Feb 19;6:1136. Originally published 2017 Jul 17. [Version 2] doi: 10.12688/f1000research.11905.2 (PMC5580409; doi:10.12688/f1000research.11905.2)
Supplement: Supplementary file 1 [file f1000research-6-15276-s0000.tgz › 6512bc55-797f-4158-a4a2-4290c09565b0.pdf]

Table S1. Results from the follow-up assay for the 31 compounds that were in the primary HTS screen: EC50 values, the final scores (active or inactive), and the ranks in the workflows 1 and 2. Partially active or single-point active compounds were considered inactives (marked by italic font). ChEMBL-NTD datasets: (N) Novartis-GNF Malaria Box (N) [1], St. Jude Children's Research Hospital Dataset (J) [2], GSK TCAMS (G) [3], DNDi HAT set (D). Compounds marked with (P) were tested in PubChem assays.

**Identifier**	**EC50 [μM]**	**Score**	**Proposed by Workflow**	**Rank (top 10'000) Workflow 1**	**Rank (top 10'000) Workflow 2**	**HTS screen**	**Known Datasets**
SJ000027935	0.29	Active	2	646	618	Active	N, J (active)
SJ000027937	0.35	Active	2	1812	678	Ambi-guous	N, J, P (active)
SJ000171111	0.36	Active	2	508	126	Active	N, J (active)
SJ000082805	0.37	Active	2	-	727	Active	N, J, G, P (active)
SJ000243624	0.41	Active	2	395	349	Active	N, J (active)
SJ000127662	0.43	Active	2	3407	490	Active	J (active)
SJ000154494	0.44	Active	1	106	-	Active	J (inactive)
SJ000140382	0.45	Active	2	-	443	Active	J (active)
SJ000147013	0.49	Active	2	4682	239	Active	
SJ000171307	0.52	Active	2	431	310	Active	N, J (active)
SJ000147025	0.57	Active	2	4431	232	Active	
SJ000180705	0.59	Active	1,2	2764	589	Active	J (active)
SJ000197041	0.62	Active	2	-	997	Active	
SJ000170251	0.85	Active	2	-	460	Ambi-guous	N, J, P (active)
SJ000243360	1.2	Active	2	-	609	Ambi-guous	
SJ000281068	1.3	Active	2	-	733	Active	J (active)
SJ000243361	1.9	Active	2	5868	450	Ambi-guous	J (active)
SJ000243592	2.0	Active	1,2	572	542	Active	J (active)
SJ000011369	2.3	Active	2	2833	534	Active	J (active)
SJ000291576	2.3	Active	2	8329	862	Active	
SJ000143975	2.3	Active	2	8797	892	Active	J, P (active)
SJ000147376	2.4	Active	2	4440	489	Active	J, P (active)
SJ000192808	2.6	Active	2	5442	958	Active	N, J (active)
SJ000144869	3.0	Active	2	4679	946	Active	J (active)
SJ000146674	3.1	Active	2	844	466	Active	
SJ000140380	3.3	Active	2	-	498	Ambi-guous	J (active)
SJ000113373	3.7	Active	2	2399	670	Active	J, D (active)
SJ000127976	3.8	Active	2	5479	926	Active	
SJ000137729	9.0	Active	1,2	474	913	Active	J (active)
SJ000143737	15.0	Active	1	6694	-	Ambi-guous	
SJ000298473		*Inactive*	1	6285	-	Inactive	

Table S2. Evaluation results for anti-malaria activity on the held-out test set (1056 molecules) for different models of Workflow 2. The maximum possible EF5% value is 10.5.

Method	AUC	EF5%
Submission (see Table 3): Linear combination of tree models and logistic regression models	0.79	4.34
Tree models	0.76	4.54
Stacked logistic regression models	0.83	4.73
Single logistic regression model	0.84	5.13

References

[1] F.-J. Gamo, L. M. Sanz, J. Vidal, C. de Cozar, E. Alvarez, J.-L. Lavandera, D. E. Vanderwall, D. V. S. Green, V. Kumar, S. Hasan, J. R. Brown, C. E. Peishoff, L. R. Cardon, J. F. Garcia-Bustos, Thousands of chemical starting points for anti-malarial lead identification. *Nature*, 465, 305-310 (2010).

[2] W. A. Guiguemde, A. A. Shelat, D. Bouck, S. Duffy, G. J. Crowther, P. H. Davis, D. C. Smithson, M. Connelly, J. Clark, F. Zhu, M. B. Jimenez-Diaz, M. S. Martinez, E. B. Wilson, A. K. Tripathi, J. Gut, E. R. Sharlow, I. Bathurst, F. El Mazouni, J. W. Fowble, I. Forquer, P. L. McGinley, S. Castro, I. Angulo-Barturen, S. Ferrer, P. J. Rosenthal, J. L. DeRisi, J. S. Lazo, D. S. Roos, M. K. Riscoe, M. A. Phillips, P. K. Rathod, W. C. Van Voorhis, V. M. Avery, R. K. Guy, Chemical genetics of *Plasmodium falciparum*, *Nature*, 465, 311-315 (2010).

[3] Novartis-GNF Malaria Box, K. Gagaring, R. Borboa, C. Francek, Z. Chen, J. Buenviaje, D. Plouffe, E. Winzeler, A. Brinker, T. Diagana, J. Taylor, R. Glynne, A. Chatterjee, K. Kuhen. Genomics Institute of the Novartis Research Foundation (GNF), 10675 John Jay Hopkins Drive, San Diego CA 92121, USA and Novartis Institute for Tropical Disease, 10 Biopolis Road, Chromos # 05-01, 138 670 Singapore.
