# Supplementary material for: Virtual-screening workflow tutorials and prospective results from the Teach-Discover-Treat competition 2014 against malaria
Source: F1000Res. 2018 Feb 19;6:1136. Originally published 2017 Jul 17. [Version 2] doi: 10.12688/f1000research.11905.2 (PMC5580409; doi:10.12688/f1000research.11905.2)
Supplement: Supplementary file 5 [file f1000research-6-15276-s0004.tgz › 9115dcd2-9a9a-4016-a633-0bdf8fb8097e.pdf]

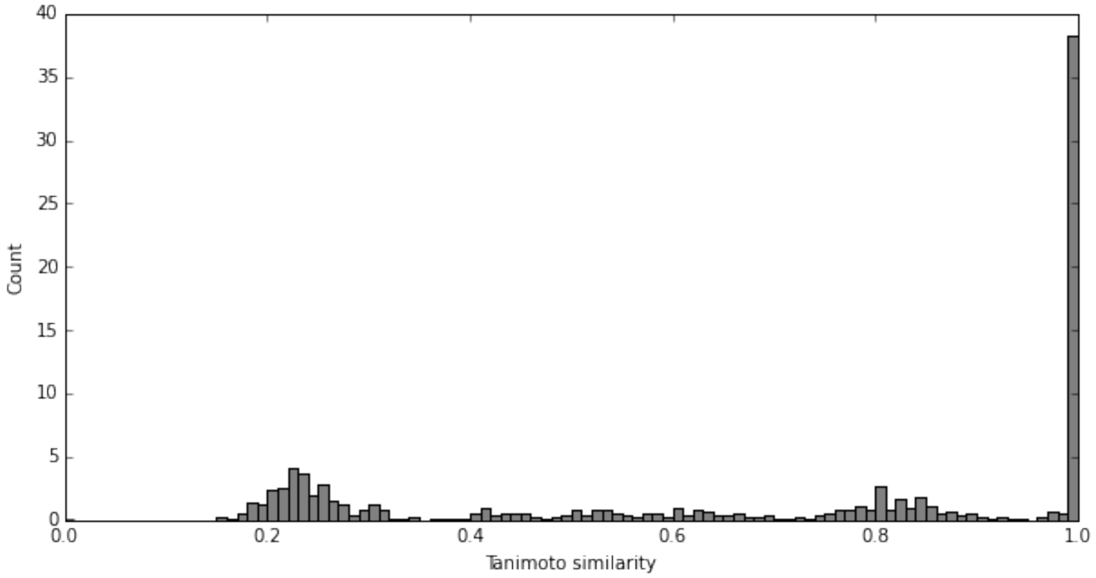

Figure S1. **Distribution between the (true) top 1000 compounds of Workflow 1 and the most similar molecule in the top 1000 list of Workflow 2.** Normalized Tanimoto similarity distribution using a Morgan2 fingerprint. 52% of the pairs have a similarity >= 0.8, 38% have a similarity of 1.0.

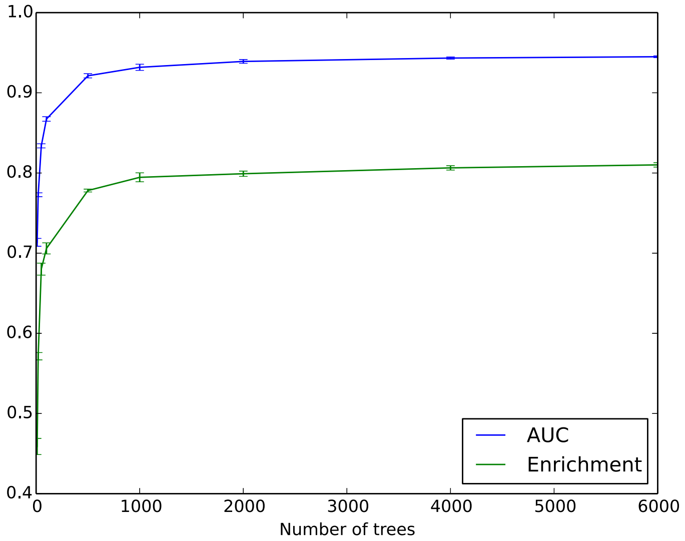

Figure S2. **Out-of-bag performance as a function of the number of trees in the ensemble models of Workflow 2.** Performance was measured by area under the ROC curve (AUC, blue) or enrichment at 5% (green).

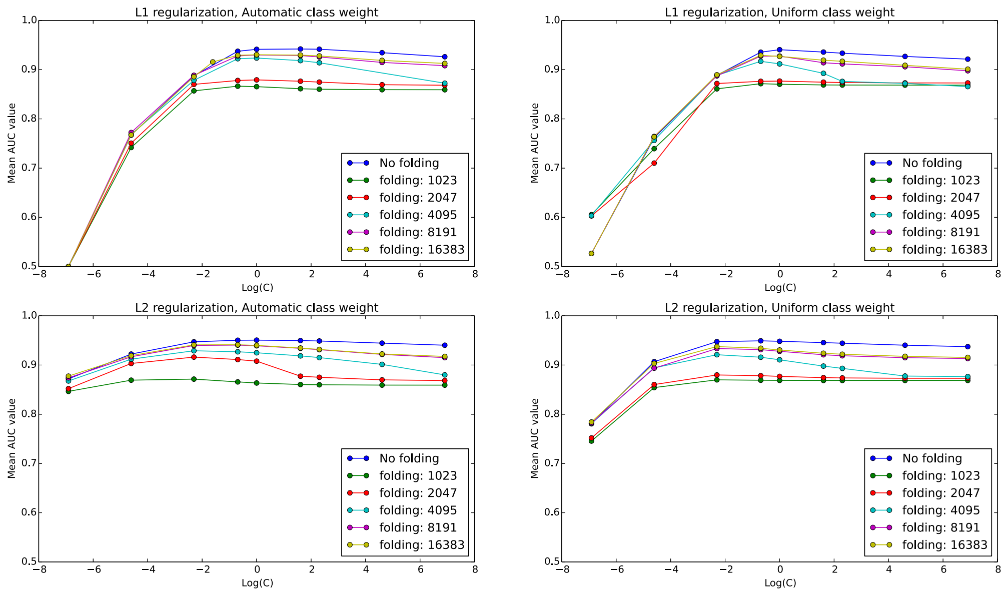

Figure S3. Parameter exploration for the logistic regression models of Workflow 2 using the area under the ROC curve (AUC). The performance on the training set as measured by the mean AUC value in a 10-fold cross-validation is given as function of the logarithm of the parameter C. C is inversely proportional to the amount of regularization in the cost function. Different colors represent different lengths for folding of the fingerprints. L1 regularization is shown in the top panels, L2 regularization in the bottom panels. Left panels use instance class weighting (to account for class imbalance), and right panels uniform class weighting.

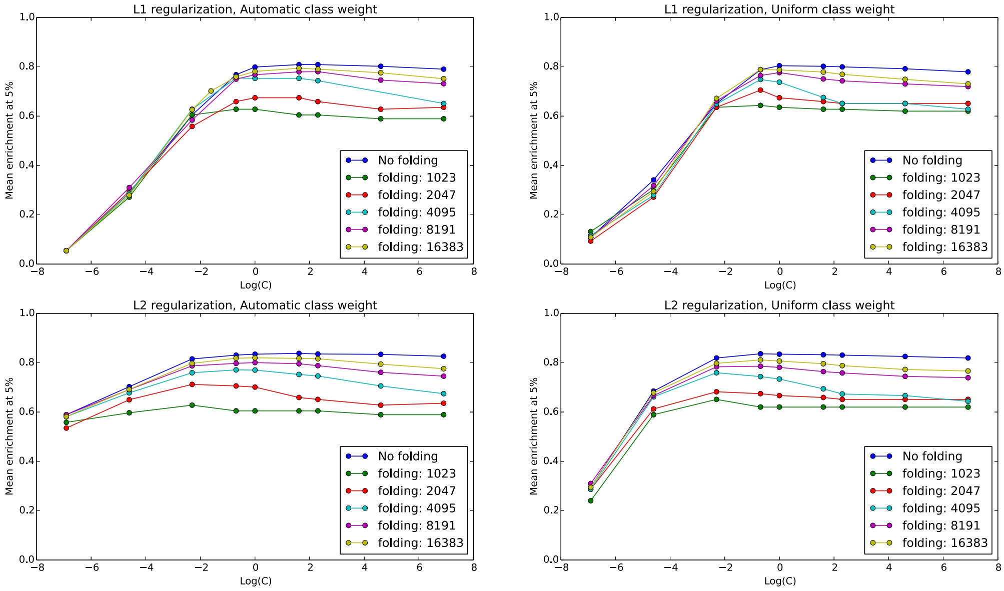

Figure S4. Parameter exploration for the logistic regression models of Workflow 2 using the enrichment at 5%. The performance on the training set as measured by the mean enrichment value in a 10-fold cross-validation is given as function of the logarithm of the parameter C. C is inversely proportional to the amount of regularization in the cost function. Different colors represent different lengths for folding of the fingerprints. L1 regularization is shown in the top panels, L2 regularization in the bottom panels. Left panels use instance class weighting (to account for class imbalance), and right panels uniform class weighting.

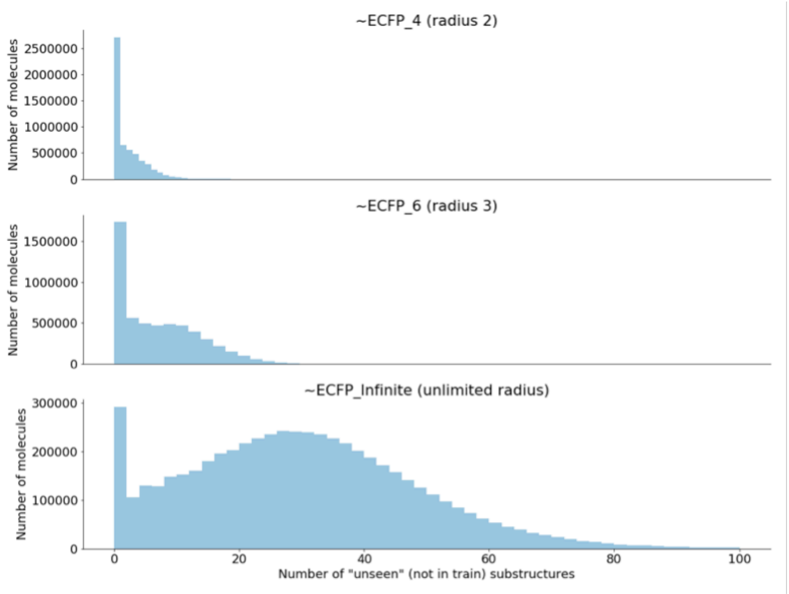

Figure S5. Number of molecules in the eMolecular catalogue as a function of the number of features in the fingerprint not present in the training set. (Top): Using a Morgan fingerprint with maximum radius = 2 (ECFP4). (Middle): Using a Morgan fingerprint with maximum radius = 3 (ECFP6). (Bottom): Using all possible circular environments in the Morgan fingerprint (unlimited radius).
