# Supplementary material for: Virtual-screening workflow tutorials and prospective results from the Teach-Discover-Treat competition 2014 against malaria
Source: F1000Res. 2018 Feb 19;6:1136. Originally published 2017 Jul 17. [Version 2] doi: 10.12688/f1000research.11905.2 (PMC5580409; doi:10.12688/f1000research.11905.2)
Supplement: Peer review history

# Peer review: Referee response for version 1

**DOI:** 10.5256/f1000research.12868.r24288

**Published:** 2017-10-24

**Authors:** Andrea Volkamer

**Affiliations:** 1 Institute of Physiology, Charité - Universitätsmedizin, Berlin, Germany

The authors introduce the two winning workflows (WFs) from the Teach-Discover-Treat competition 2014 featuring ligand-based virtual screening pipelines for anti-malaria compounds together with results from an experimental follow-up study. The workflows show hit rates of 57% (52% WF1, 100% WF2) in a prospective study on a rather small sample size due to compound availability and funding reasons (72 WF1/ 9 WF2 novel compounds).  The article is well written, easy to understand and the study showed promising results in finding new active compounds. Furthermore, both workflows are available to the community.

 Minor comments that could be addressed to improve the manuscript:

 Methods/Workflows:

 - More detail on the more advanced ML techniques, number of features, and especially feature importances would be helpful for the reader.

 - The data set is highly unbalanced (~1.5K actives vs. 290K inactives). Could one expect a boost in performance when using under-/oversampling methods?

  - This may have been addressed in the competition itself, but it would be interesting to see how a simple model performs on the data, for example simply ranking by similarity to known actives?

 Evaluation:

 - The authors admit the little flaw in the original WF1 that the top 1000 molecules accidently represent a random selection from the top 10K. It’s hard to compare the results now that the selection and testing phase is over, but it would be nice to see some evidence that the intended selection strategy would actually have been superior. E.g. the positions of the intended ranking could be included in Table 5, the prospectively tested set is small but a trend may become apparent?

 - Since 31 of the selected 114 compounds (~30%) were present in the HTS, I’m wondering how many of the HTS compounds were present in eMolecules in total?  Because they have been used for training, taking them out of the evaluation would be more convincing and would probably also improve the rankings of the tested compounds.  

 - The authors claim that the two methods pick generally similar compounds, which can be somehow expected from the design of the two WFs (similar MLs and fingerprints). Nevertheless, this trend is not obvious to me from the few mentioned values, e.g., 7 compounds selected from WF2 are in top 10K from WF1 (page 8). It would be more meaningful to calculate the overlap of the top 1000 compounds between the methods or the similarity between these compounds (also with respect to different top 1000 selections in WF1, see point above).

I have read this submission. I believe that I have an appropriate level of expertise to confirm that it is of an acceptable scientific standard.

# Response

**Published:** 2018-02-13

**Authors:** Sereina Riniker

**Affiliations:** ETH Zürich, Switzerland

We follow the order of the points raised by the reviewer. 

- For both workflows, the code is freely available on GitHub (URLs are given in the paper).
- Dataset imbalance: Workflow 1 uses an undersampling method for the random forest to deal with this issue. We will add a sentence describing this in the revised version. No over- or undersampling method was used for the models in Workflow 2. However, the logistic regression models did include an instance weighting scheme that gives more weight to instances of the minority class (i.e. C is modified per sample proportionally to the proportion of its class in the dataset). We will add in the Supplementary Material to show that class imbalance correction did not influence the model performance in Workflow 2.
- To our knowledge this comparison has not been done in the competition itself for the external held-out set. In Task 2 of Workflow 1, the performance of the ML models is compared to simple ranking by similarity (see Table 2). The latter showed already a good baseline performance such that only random forest models were able to outperform it. We will add a sentence discussing this.
- The question is what the comparison would be to define superiority without results for all compounds. Ideally, the enrichment in the selected 1000 compounds versus in the direct top 1000 should be compared but that is not possible anymore at this stage. It is important to stress that the selection procedure was aimed at improving the SAR information in the selected 1000 compounds not necessarily at increasing the hit rate, because the top 10'000 are already the highest ranked compounds among 5.5 millions in eMolecules. We will add a sentence regarding this in the revised version. We will replace column "Rank (1000) Workflow 1" in Table 5 with a column "Proposed by Workflow" to provide the information about which workflow proposed which molecules. Further, we will add a separate file in the Supplementary Material with the 1000 molecules selected by the correct Workflow 1. 
- We agree with the reviewer that removing the HTS compounds from the eMolecules catalogue prior to ranking should have been done, but for the present work it is unfortunately too late.
- 52% of the (true) top 1000 compounds of Workflow 1 have a similar compound in the top 1000 of Workflow 2, using Morgan2 fingerprints (radius = 2, 4096 bits) and a Tanimoto similarity cut-off = 0.8. We will add a figure with the distribution of the similarity value between each compound of Workflow 1 and its most similar compound in Workflow 2 in the Supplementary Material together with a short discussion in the main text. 

We thank the reviewer for carefully reading the manuscript and for the constructive and insightful feedback.

# Peer review: Referee response for version 1

**DOI:** 10.5256/f1000research.12868.r24675

**Published:** 2017-08-16

**Authors:** Matthew P. Baumgartner

**Affiliations:** 1 Eli Lilly and Company, Windlesham, UK

The authors report on their participation in the 2014 Teach-Discover-Treat (TDT) initiative. The goal of the TDT is to encourage the creation of practical tutorials for computational chemistry. The authors present the two workflow tutorials that they developed for Challenge 1 of the competition. The challenge involved three tasks: analyzing single-point phenotypic HTS results and follow-up dose-response data for a subset of the compounds, building a predictive model of the anti-malaria activity and using that predictive model to select compounds from a set of commercially available compounds for prospective testing. The first workflow presented by the authors only used the HTS data for its predictions and the second used both the HTS and dose-response data. 

 Overall I think that the paper is a thorough and easy-to-follow description of the methods and results of the two workflows, but there a few items that I feel require revisions. 

 Page 5, a brief description of what "heterogeneous classifier fusion" is would be appreciated

 Page 6. The authors should list the total number of features that they use as descriptors in workflow 2. 

 Page 6. When building the random forests and extremely randomized trees of varying sizes, the ensembles of trees with 6000 trees (the largest number tested) were shown to preform best. The authors should explain why they did not try a higher number of trees. 

 Page 6. In the "Task 2..." paragraph. The authors should state what the resulting linear combination of the models was. The ratio would be interesting to know.

 Page 8, in the paragraph starting "The results for the remaining...".  It states in the text that there were 9 compounds predicted by workflow 2 that tested, but in Table 5, there are 10 compounds from workflow 2 listed. This should be corrected or clarified. 

 Page 8 and Table 5. As the compounds from Workflow 1 were selected randomly due to an error, is it meaningful to list their rankings at all?

I have read this submission. I believe that I have an appropriate level of expertise to confirm that it is of an acceptable scientific standard.

# Response

**Published:** 2018-02-13

**Authors:** Sereina Riniker

**Affiliations:** ETH Zürich, Switzerland

We follow the order of the points raised by the reviewer.

- We will add a sentence about "heterogeneous classifier fusion" in the revised paper, including a reference to Ref. 13, which contains a detailed discussion of this concept.
- Descriptors in Workflow 2: The RDKit descriptor set consists of 196 features and the unfolded fingerprints set, after removal of redundant features, 1'265'410 different substructures. We will add this information in the revised version.
- Workflow 2: Larger numbers of trees were not investigated due to limited computer resources. However, a plateau in the performance curve was observed after 2000 trees, thus only small improvement can be expected for models with more than 6000 trees. We will add a figure to the Supplementary Material.
- Workflow 2, Task 2: The linear combination in Workflow 2 placed substantially more weight on the tree models (coefficient 1.07) than on the logistic regression models (coefficient 0.07). We found later that this weighting was not optimal for the prediction of the held-out test set. Logistic regression models alone would have performed better than the original submission from Workflow 2. We will add a table with this information in the Supplementary Material and a comment in the main text.
- There were nine new compounds and one known anti-malarial amodiaguine, i.e. together ten compounds. We will add the term "new" in one sentence of the corresponding paragraph to make it clearer.
- Table 5: We agree that they are not true rankings anymore, but because this list served as the input for compound selection the rankings are used in Table 5 to mark which molecules came from Workflow 1. We will replace column "Rank (1000) Workflow 1" with a column "Proposed by Workflow" to provide the same information.

We thank the reviewer for carefully reading the manuscript and for the constructive and insightful feedback.

# Peer review: Referee response for version 1

**DOI:** 10.5256/f1000research.12868.r24285

**Published:** 2017-08-02

**Authors:** David Ryan Koes

**Affiliations:** 1 Department of Computational and Systems Biology, University of Pittsburgh, Pittsburgh, PA, USA

The report describes the two winning ligand-based virtual screening methods of the 2014 Teach-Discover-Treat exercise.  Both workflows and the supporting data are available in their entirety online (behind a request for the user's email address) and are adequately described in the manuscript.  The report is of general interest to the community and a useful resource to practitioners in ligand-based drug discovery.

 I have a few minor suggestions for strengthening the manuscript.

 Page 4. A few sentences describing "heterogeneous classifier fusion" would be appreciated.

 Page 5.  Descriptors. I would be interested in knowing the number of bits (i.e. unique ECFP/FCFP fragments) required to represent the full dataset (that is, the number of features in the input, which I suspect is actually larger than the number of examples?).

 Page 5. Task 2.  The weights for the two models found by the linear regression would be interesting to report (is one model favored more heavily than the other?).

 Table 4.  This would be a bit more informative if variance was reported as well.

 Figure 2.  It isn't clear to me exactly what this is reporting.  Is this the distribution of all possible pairs between the two sets? Please clarify.

 Table 5.  My understanding is that the Rank (1000) numbers are essentially meaningless as the compounds were (accidentally) randomly selected.  Can the corrected top 1000 ranks  be provided as well (or instead) and clearly labeled as such (realizing that not all compounds will have such a rank).  

 It's also hard to get a sense of enrichment from these numbers since only 114 compounds were tested but the ranks have a much larger span.  For example, the workflow 2 active compounds have poor ranks (>500), but this is misleading since there were no highly ranked (novel) compounds tested.  I would really appreciate some visualization of enrichment relative to ranking (e.g. ROC curve) for 114/81 compounds tested for workflow 1.

I have read this submission. I believe that I have an appropriate level of expertise to confirm that it is of an acceptable scientific standard.

# Response

**Published:** 2018-02-13

**Authors:** Sereina Riniker

**Affiliations:** ETH Zürich, Switzerland

We follow the order of the points raised by the reviewer.

- We will add a sentence about "heterogeneous classifier fusion" in the revised version, including a reference to Ref. 13, which contains a detailed discussion of this concept.
- Descriptors in Workflow 1: The actives in the primary HTS set yield 5935 unique Morgan2 fragments. Together with the inactives there are 56'351 unique fragments. The number of 4096 bits used for the folded Morgan2 fingerprints in Workflow 1 is clearly much smaller than the number of unique fragments, however, a balance must be found between the size of the fingerprint (and associated computational cost) and the number of collisions. In our case, we found that a size of 4096 bits presents a good compromise.
- Workflow 1, Task 2: The weights of all models in the classifier fusion of Workflow 1 were the same. The MAX rank was used.
- We will add the standard deviation to Table 4.
- Figure 2 reports the distribution of all possible pairs between the two sets. We will adapt the legend of Figure 2 to make it clearer.
- Table 5: As the list with ranks served as the input for compound selection, the rankings are used in Table 5 to mark which molecules came from Workflow 1. We will replace column "Rank (1000) Workflow 1" with a column "Proposed by Workflow" to provide the same information. We will also add a separate file in the Supplementary Material with the 1000 molecules selected by the correct Workflow 1.
- Both enrichment factors and ROC curves compare the ranking of active/inactive molecules against random distribution. The number of actives is exceptionally high in the set of 81 compounds (i.e. 58 %), because these were selected among the highest-ranked compounds from the 5.5 millions in eMolecules. Due to this high percentage of actives in the list, the calculation of enrichment factors or ROC curves does not make much sense in the present case.

We thank the reviewer for carefully reading the manuscript and for the constructive and insightful feedback.
